# A New Approach to the Synthesis of Nanocrystalline Cobalt Boride in the Course of the Thermal Decomposition of Cobalt Complexes [Co(DMF)_6_]^2+^ with Boron Cluster Anions

**DOI:** 10.3390/molecules28010453

**Published:** 2023-01-03

**Authors:** Elena A. Malinina, Ivan I. Myshletsov, Grigorii A. Buzanov, Alexey S. Kubasov, Irina V. Kozerozhets, Lyudmila V. Goeva, Svetlana E. Nikiforova, Varvara V. Avdeeva, Konstantin Yu. Zhizhin, Nikolay T. Kuznetsov

**Affiliations:** Kurnakov Institute of General and Inorganic Chemistry, Russian Academy of Sciences, Moscow 119991, Russia

**Keywords:** cobalt boride, boron nitride, boron cluster anion, precursor, thermal decomposition

## Abstract

In the course of the study, nanocrystalline cobalt monoboride was prepared by thermal decomposition of precursors [Co(DMF)_6_][An], where [An] = [B_12_H_12_]^2−^ (**1**), [*trans*-B_20_H_18_]^2−^ (**2**) or [B_10_Cl_10_]^2−^ (**3**) in an argon atmosphere. Three new salt-like compounds **1**–**3** were prepared when Co(NO_3_)_2_ was allowed to react with (Et_3_NH)_2_[An]. Compound **1** is new; the structures of compounds **2** and **3** have been previously reported. Samples **1**–**3** were annealed at 900 °C in argon to form samples **1a**–**3a**, which were characterized by single crystal XRD for **1** and powder XRD for **1–3**. Powder XRD on the products showed the formation of BN and CoB for **1a** in a 1:1 ratio; **2a** gave a higher CoB:BN ratio but an overall decreased crystallinity. For **3a**, only CoB was found. IR spectra of samples **1a**–**3a** as well as X-ray spectral fluorescence analysis for 3a confirmed these results. The nanoparticular character of the decomposition products **1a**–**3a** was shown using TEM; quite small particle sizes of about 10–15 nm and a quite normal size distribution were found for **1a** and **2**a, while the decomposition of **3** gave large particles with 200–350 nm and a broad distribution.

## 1. Introduction

The preparation of high purity nanocrystalline metal borides has attracted the attention of many material chemists due to the remarkable physicochemical properties of these compounds, such as high strength, refractoriness, resistance to oxidation, corrosion, wear, etc. [1,2,3,4]. Cobalt borides are compounds with the general formula Co*_x_*B*_y_*; two main representatives are CoB and Co_2_B. It is known that cobalt(II) borides exhibit excellent catalytic activity in the production of hydrogen [5,6,7,8,9] and oxygen [7,10,11,12,13], in the liquid phase hydrogenation of citral [14,15], and can be used as anode materials in batteries [16,17].

To date, transition metal borides are obtained, as a rule, in the form of amorphous powders, which can lead to the presence of impurities and defects in the final material. Among the known methods for the preparation of nanoparticles of cobalt(II) borides Co*_x_*B, the most common is the chemical reduction of cobalt(II) chloride with alkali metal tetrahydroborate [5,9,13,17,18,19,20]. In addition, cobalt(II) borides Co_2_B, CoB can be prepared by the interaction of cobalt(II) chloride or metal with elemental boron [21]. Moreover, preparation of mesoporous nanocrystalline α-CoB_x_ was synthesized by chemical reduction of cobalt acetate with dimethylamine borane [20].

One of the possible ways to stabilize nanoparticles is the use of cages capable of encapsulating metal borides. In particular, a framework of hexagonal boron nitride is capable of encapsulating cobalt borides [10,22,23]. One of the possible ways to obtain metal boride nanoparticles in a similar way is based on the use of thermal plasma [24]. It should be noted that this method is a multi-stage process and requires high costs, both economic and time. In addition, laser irradiation, magnetron sputtering, γ-radiolysis, and other methods can be noted; see details in recent review [25].

The development of alternative, simple, and convenient methods for the preparation of high purity transition metal boride nanoparticles is still an urgent problem in applied chemistry. We suggested that metal-containing boron clusters [26,27,28,29,30,31] can be used as sources to prepare metal borides. These power-intense compounds can lower the temperatures need to form final products. Our preliminary studies showed that moderate heating of nickel(II) and cobalt(II) complexes with the *closo*-decaborate anion [M(solv)_6_][B_10_H_10_] (M = Co, Ni; solv = H_2_O, N_2_H_4_, DMF, DMSO) [32,33,34,35] resulted in boron-oxide phase and metal borides (the conclusions were based on IR spectroscopy data of amorphous samples). X-ray powder diffraction studies allowed us to conclude that solid solutions Ni_3_C_1 –*x*_B*_x_* and Ni_3_C were formed when annealing [Ni(DMF)_6_][B_10_H_10_]; BN and Co_2_B phases were detected when annealing [Co(N_2_H_4_)_3_][B_10_H_10_]. Encouraging with the fact that formation of boride and boron-oxide phases can be suggested at thermal heating of [Co(DMF)_6_][B_10_H_10_] at 650 °C [32], which was concluded based on IR spectroscopy, we decided to study this process in detail and determine the effect of changing the boron cluster on composition and properties of final products. In order to avoid preparation of boron-oxide phases which can be polyborates [36], we studied similar compounds with less reactive boron clusters as compared to the *closo*-decaborate anion, namely, [B_12_H_12_]^2−^, [*trans*-B_20_H_18_]^2−^ and [B_10_Cl_10_]^2−^.

In this work, we propose a low-temperature method for the preparation of nanocrystalline cobalt monoboride based on the thermal decomposition of cobalt(II) coordination compounds with energy-intensive boron cluster anions. As started compounds we used complexes [Co(DMF)_6_]An (An = [B_12_H_12_]^2−^, [*trans*-B_20_H_18_]^2−^ and [B_10_Cl_10_]^2−^) to determine the effect of the number of boron atoms per metal atom on the composition of the final products.

## 2. Results and Discussion

### 2.1. Synthesis of Precursors [Co(DMF)_6_][An]

**Scheme 1:**
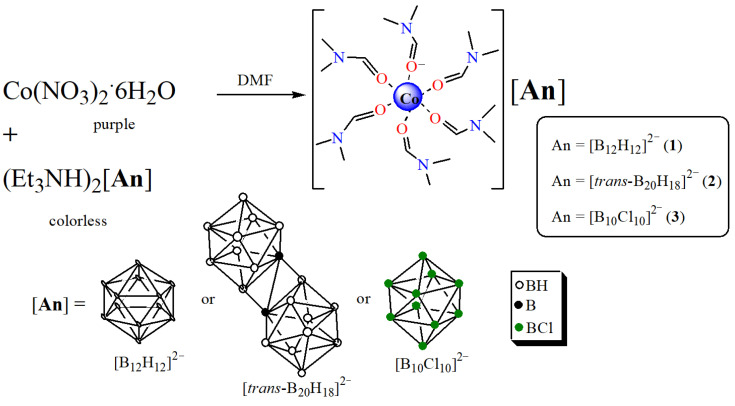
Synthesis of precursors **1**–**3**.

In this work, we used cobalt(II) complexes with coordinated molecules of *N,N*-dimethylformamide, which are easily evaporating groups, as precursors [Co(DMF)_6_][An]. The starting complexes were obtained by reacting cobalt(II) nitrate with the corresponding triethylammonium salts of boron cluster anions in DMF; the final compounds precipitated from the reaction mixture as purple crystals (Scheme 1).

The resulting compounds **1, 2**∙3H_2_O, and **3** were identified by elemental analysis, IR spectroscopy, and X-ray diffraction. The structures of [Co(DMF)_6_][B_10_Cl_10_] (**1**) [37] and [Co(DMF)_6_][B_20_H_18_] (**2**) [38] are known: water-free compound **2** was obtained when anhydrous CoCl_2_ was allowed to react with [B_20_H_18_]^2−^; for **1** and **3**, water-free samples were isolated when using cobalt nitrate crystal hydrate. The structure of [Co(DMF)_6_][B_12_H_12_] (**1**) was determined by X-ray diffraction in this paper.

The IR spectra of **1** and **2**∙3H_2_O contain an intense absorption band in the region of 2550–2400 cm^−1^, which is related to ν(BH) of the boron cluster anions [B_12_H_12_]^2−^ and [*trans*-B_20_H_18_]^2−^ (Figures S1a,b). In the IR spectrum of **3**, bands ν(BCl) are observed at 1157, 1002 cm^−1^; a band of stretching and bending vibrations of the B–B groups in boron cage is observed near 843 cm^−1^ (Figure S1c).

The coordinated state of DMF molecules by the oxygen atom of the carbonyl group is evidenced by the shift of band ν(C=O) (1651, 1664, and 1652 cm^−1^ in the IR spectra of **1**, **2∙**3H_2_O and **3**, respectively) into a lower frequency interval compared to that in the spectrum of free DMF (ν(C=O) is 1679 cm^−1^). It should be noted that the IR spectrum of **2∙**3H_2_O contains ν(OH) at about 3425 cm^−1^, which is characteristic of associated water molecules. The IR spectra of **1** and **3** contain no bands in this region, thus indicating the formation of water-free compounds.

**Figure 1:**
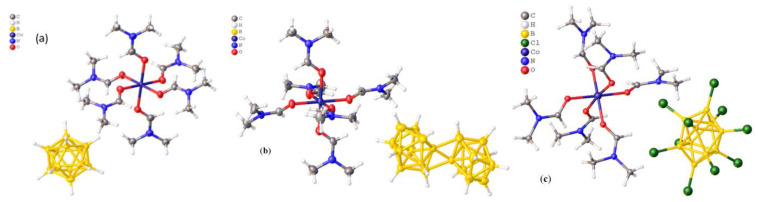
Structures of samples (**a**) **1**; (**b**) **2**; (**c**) **3**.

According to single-crystal X-ray diffraction data, crystal **1** is built of cobalt(II) complexes [Co(DMF)_6_]^2+^ and anions [B_12_H_12_]^2−^ (Figure 1a). The triclinic unit cell (space group *P*-1) of compound **1** contains four ½ complex cations located at the inversion centers and two anions occupying a common position. The coordination environment of Co(II) atoms includes six oxygen atoms from six DMF molecules. The octahedra of metal atoms are slightly distorted. The Co–O bond lengths are in the range of 2.067–2.102 Å in **1**. The O–Co–O bond angles are in the range of 87.2–92.8° and 87.6–92.4°, respectively. In the packing of the structure of **1**, complex cations [Co(DMF)_6_]^2+^ form three-dimensional networks with square channels directed along axis *a* (Figure S2). The [B_12_H_12_]^2−^ anions are located in the center of these channels.

The structures of samples **2** [38] and **3 [37]** were determined early; they are structurally similar and are shown in Figure 1b,c. Note that water-free sample **2** was isolated when anhydrous CoCl_2_ was allowed to react with (Et_3_NH)_2_[B_20_H_18_]; here we used sample **2**∙3H_2_O.

**Figure 2:**
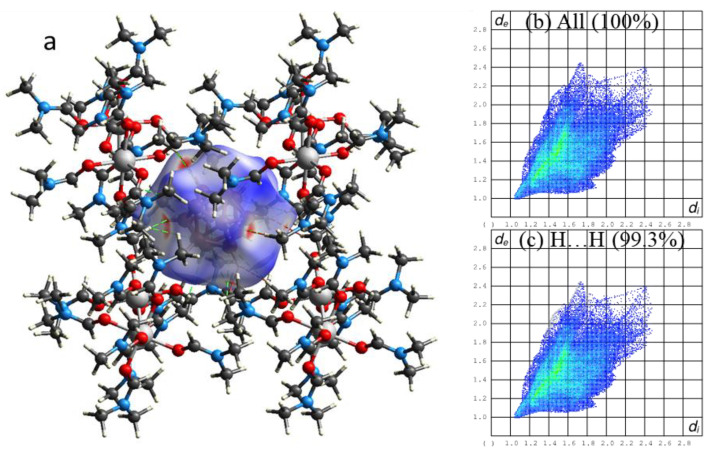
(**a**) *d*_norm_ surface of compound **1**∙, (**b**) full two-dimensional fingerprint plot for the [B_12_H_12_]^2−^ anion together with that delineated into H…H (**c**) contacts.

Hirshfeld surface analysis was performed for compound **1**. The [B_12_H_12_]^2−^ anions are surrounded by eight [Co(DMF)_6_]^2+^ cations; weak CH…BH interactions between the cations and anions are observed (in Figure 2a, dotted green lines show the B…H contacts, dotted red lines show the H…H contacts, the distances between atoms in which are less than the sum of the van der Waals radii). The strongest CH…BH interactions are represented on the Hirschfeld surface of the anion as red spots. Analysis of the full two-dimensional fingerprint plot shows that the H…H contacts of the anion account for 99.3% of the surface.

X-ray powder diffraction data revealed that samples **1**, **2∙**3H_2_O, and **3** are individual compounds and contain no impurities (Figure S3). Experimental X-ray diffraction patterns for **1**, **2∙**3H_2_O, and **3** were compared to calculated data found from single-crystal X-ray diffraction studies of compounds **1**–**3**. Some discrepancy in the X-ray diffraction pattern of **2** and **2∙**3H_2_O can be explained by the presence of associated water molecules in sample **2∙**3H_2_O.

**Figure 3:**
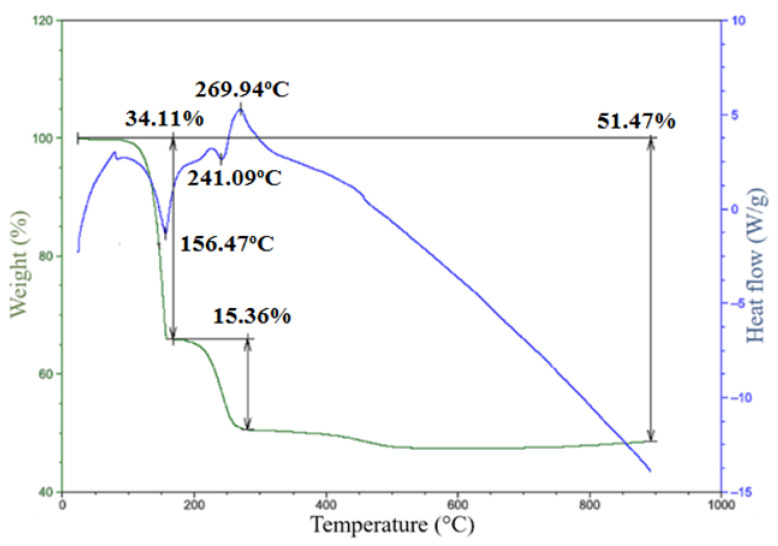
Thermogram of **1**.

TGA studies of samples [Co(DMF)_6_][An] (**1**–**3**) were performed in argon. Sample **1** is stable up to 156.47 °C (Figure 3). At this temperature, an endothermic effect appears on the thermogram, accompanied by a loss of sample weight of 34.11% which corresponds to loss of three DMF molecules and corresponding to the first stage of the removal of the organic part of the complex. With an increase in temperature to 241 °C, the second endothermic effect is observed on the thermogram, accompanied by a weight loss of the sample of 15.36% which corresponds to one DMF molecule. A further increase in temperature leads to an exothermic effects at 241.09 °C, which is accompanied with a weight loss of 51.47% and corresponds to loss of two remaining DMF molecules. An exothermic effect at 269.94 °C is accompanied with no weight loss and can be assigned to the opening of the *closo*-structure of the boron cluster to form *nido*-structures; an effect near 460 °C without weight loss can be assigned to rearrangement of *nido*-system and further destruction of the boron cage.

**Figure 4:**
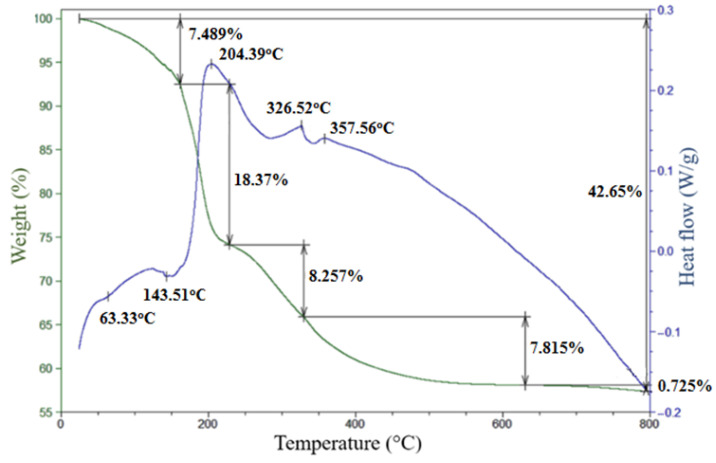
Thermogram of **2**∙*n*H_2_O.

For **2**∙3H_2_O, a gradual weight loss of 7.489% is observed in the temperature range of 20–160 °C, which is accompanied by a broadened endothermic effect with a final value of 143.5 °C (Figure 4). These processes correspond to the removal of three associated water molecules, which is consistent with the data of the IR spectrum of **2**∙3H_2_O, where the ν(OH) stretching vibration band characteristic of associated water molecules is observed at 3450 cm^−1^.

In the range of 155–220 °C, an exothermic effect is observed with a maximum at 204 °C, which is accompanied by a weight loss of 18.37% and corresponds to the removal of two coordinated DMF molecules. With a further increase in temperature, an exothermic effect is observed at 326 °C with sample weight loss of 8.257%, which corresponds to the removal of another DMF molecule. The broadened exothermic effects at 357 °C without weight loss corresponds to the opening of the *closo*-system of the boron cage; an effect at 470 °C can be assigned to rearrangement of the *nido*-system of the boron cage. Further heating results in gradual destruction of the boron cage accompanied by a weight loss of 7.815 °C of the sample which corresponds to release of one DMF molecule.

**Figure 5:**
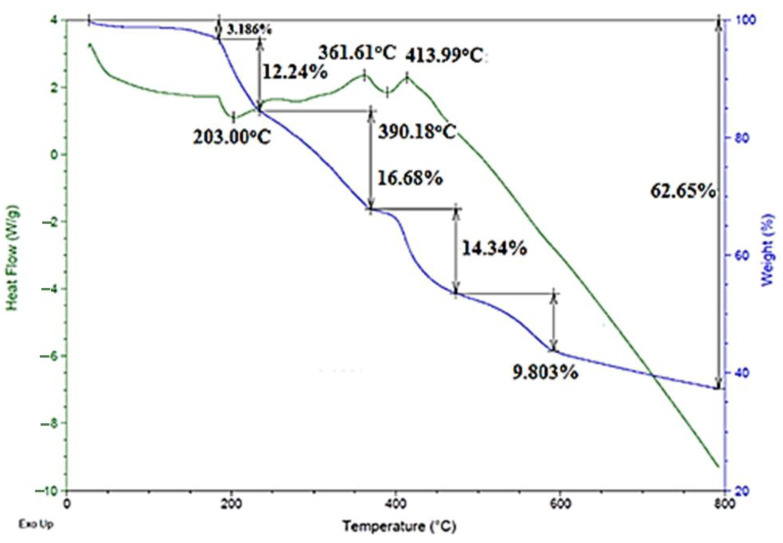
Thermogram of **3**.

Three pronounced thermal effects are observed on the thermogram of sample **3** (Figure 5). The endothermic effect with a maximum at 203 °C, accompanied by a loss of sample weight 15.43%, corresponds to the removal of two DMF molecules. Two exothermic effects at 361.61 and 413.99 °C, accompanied by a weight loss of 16.68 and 14.34%, respectively, indicate the stepwise removal of four DMF molecules with simultaneous destruction of the *closo*-system of the boron cage. With further heating of the sample above 500 °C, no thermal effects are observed on the differential curve; however, the TG curve shows a mass loss of 9.803%, corresponding to the partial removal of exopolyhedral chlorine atoms with the formation of a Cl_2_ molecule.

Based on the results obtained, the annealing temperature needed to prepare boride phases was determined to be 900 °C in order to achieve maximum structuration of samples. When heated **1**, **2**∙3H_2_O, and **3** at 900 °C in argon, products **1a**, **2a,** and **3a**, respectively, were prepared.

### 2.2. Thermal Decomposition of **1**–**3**

**Figure 6:**
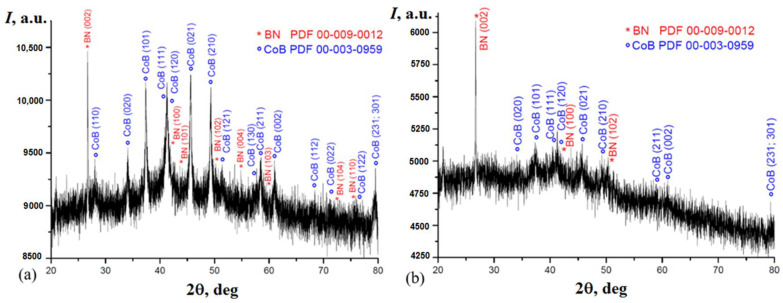
X-ray powder diffraction pattern for samples (**a**) **1a** and (**b**) **2a**.

The completeness of annealing of precursors **1**–**3** and the phase composition of samples **1a**–**3a** were determined based on X-ray powder diffraction and IR spectroscopy data. On the X-ray powder diffraction patterns of the annealed samples, visually distinguishable reflections of the observed crystalline phases are noted and the corresponding Miller indices are marked (Figure 6). The card numbers of the PDF-2 X-ray powder database used in this work are shown.

According to X-ray powder diffraction data, the annealing product **1a** is a two-phase mixture (Figure 6a). The diffraction pattern contains reflections corresponding to boron nitride BN in the hexagonal modification, as well as reflections of cobalt monoboride CoB.

In the case of sample **2a**, boron nitride reflections (hexagonal modification) are also observed in the diffraction pattern (Figure 6b). However, in contrast to **1a**, the relative intensity of the reflections is lower, which can be caused by a decrease in the particle size of the CoB phase. Apparently, the increase in the size of CoB crystallites is hindered by their dilution in the nitride matrix due to an increase in the ratio of the number of boron atoms in the cluster (12 versus 20 boron atoms) to the number of nitrogen atoms in DMF molecules (compared with sample **1a**).

**Figure 7:**
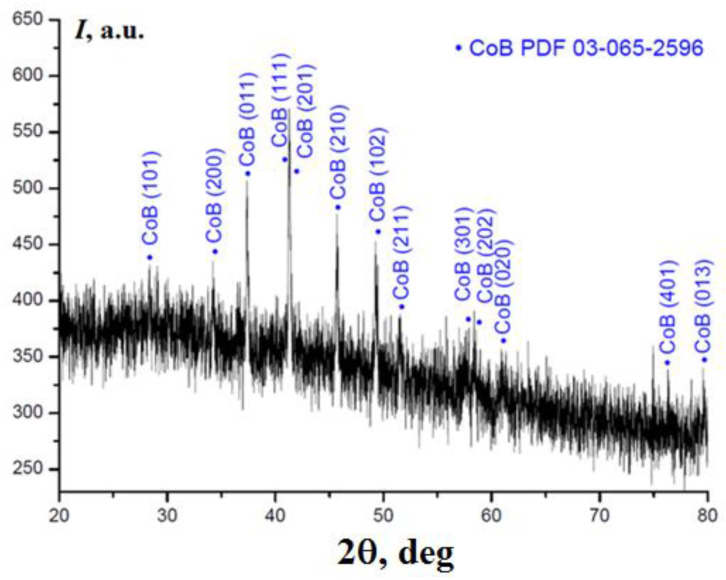
X-ray powder diffraction pattern for sample **3a**.

The diffraction pattern of sample **3a** shows reflections of the CoB crystalline phase (Figure 7) without other reflections.

The formation of cobalt monoboride CoB in the tetragonal modification is observed for all samples; for **1a** and **2a**, it is better described by the space group *Pbnm*, the annealing product **3a** has a crystal lattice with the space group *Pnma*.

In the case of all studied annealed compounds, the formation of other phases of the Co–B system (other cobalt borides, metallic cobalt, or crystalline boron) was not detected by X-ray powder diffraction. However, the presence of some amorphous components is expected.

The absence of absorption bands of stretching vibrations ν(BH) at 2466 cm^−1^ and 2532, 2497 cm^−1^ in the IR spectra of samples **1a** and **2a**, respectively, or ν(BCl) at 1157, 1002, and ν + δ(BB) at 843 cm^−1^ in the IR spectrum of **3a**, as well as ν(C=O) at 1651, 1664, and 1652 cm^−1^ indicates complete destruction of boron cluster anions and coordinated dimethylformamide molecules, respectively (Figures S4–S6). The absorption band at 780 cm^−1^ in the IR spectrum of **1a** and 781 cm^−1^ in the IR spectrum of **2a**, which, according to the literature data [39], refers to stretching vibrations of the ν(BN) bond, indicates the formation of a boron nitride phase in annealed samples **1a** and **2a**. It should be noted that this absorption band is not observed in the IR spectrum of the annealed sample **3a** (Figure S6). In addition, the presence of absorption bands at ~1100 and ~798 cm^−1^, as well as the correlation with the IR spectrum of amorphous boron, suggests the presence of an amorphous boron phase in annealed samples **1a**–**3a**. It seems logical because using the started samples, the Co:B ratio is 1:12, 1:20, and 1:10 for **1**, **2∙**3H_2_O, and **3**, respectively. In the products, CoB and BN are observed (note that Co:N ratio is 1:6 for all the starting samples). Therefore, formation of amorphous boron is expected for all three samples.

Analyzing the data obtained from TGA studies, we can discuss the composition of compounds formed in samples **1a**–**3a**. Note that the presence of BN in products 1a and 2a indicate that DMF molecules release with their decomposition and N atoms reacts with B atoms. Starting from [Co(DMF)_6_][B_12_H_12_] (**1**, M = 639.90 g/mol), we can assume that sample **1a** is a mixture of CoB + 6 BN + 5 B (M = 273.85 g/mol), which corresponds to total weight loss of 57.35 % (calcd.) which close to 51.47% (from TGA data). Thermal decomposition of sample [Co(DMF)_6_][B_20_H_18_]∙3H_2_O (**2**∙3H_2_O, M = 785.85 g/mol) results in CoB + 6 BN + 13 B, the same calculations give us the total weight loss of 45.81% (calcd.) vs. 57.35% (from TGA data). The discrepancy in experimental and theoretical data is explained further weight loss of the sample after 800 °C. Annealing of [Co(DMF)_6_][B_10_Cl_10_] (**3**, M = 960.13 g/mol) gives us the composition of sample **3** to be CoB + 6 BCl + 3 B (379.75 g/mol); the calculated total weight loss is 39.50% which is very close to 37.35% (calculated from TGA data).

### 2.3. Morphology of Samples **1a**–**3a**

**Figure 8:**
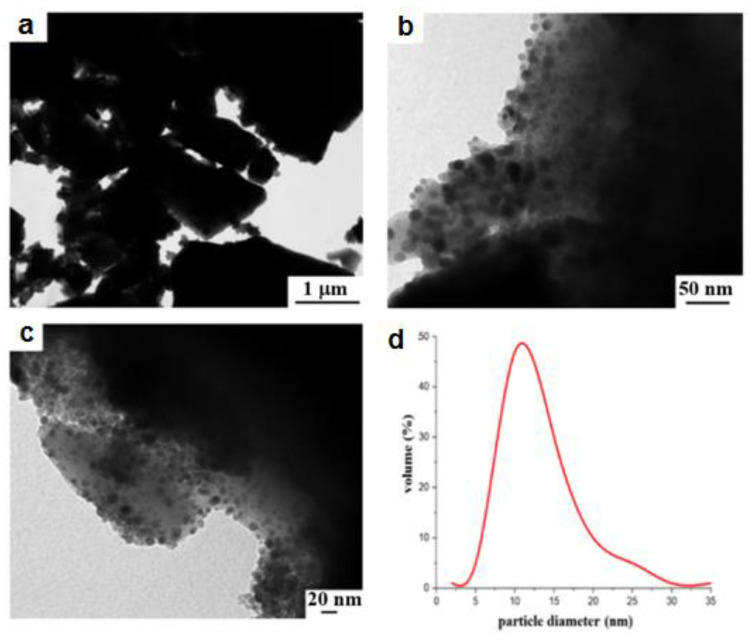
(**a**–**c**) TEM images and (**d**) particle size distribution curve of sample **1a**.

According to the presented TEM images (Figure 8), sample **1** is an inhomogeneous two-component system, which consists of large blocks and nanosized particles of cobalt monoboride crystallized on its surface, which have isometric shape. As can be seen from the distribution curve, the sizes of isometric particles are in the range of 2–35 nm. The average nanoparticle size is 11 nm (Figure 8d).

**Figure 9:**
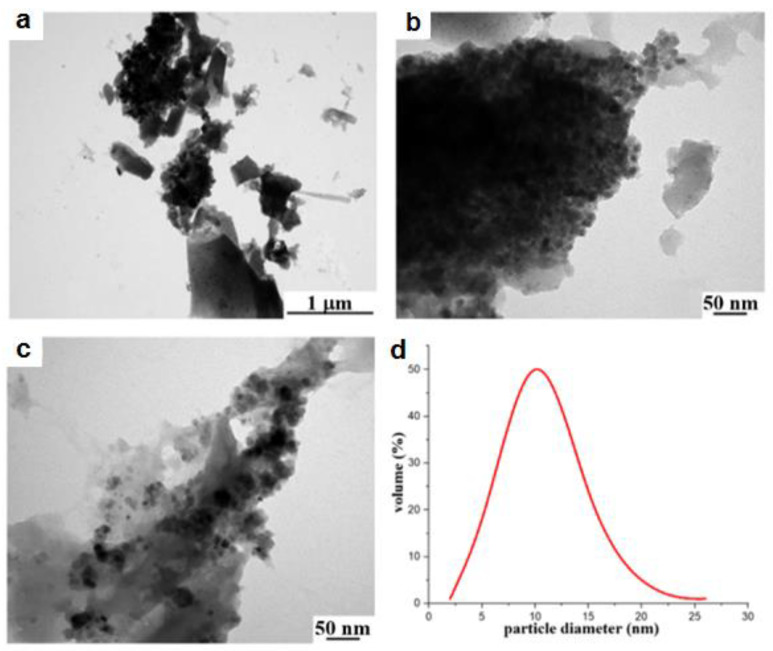
(**a**–**c**) TEM images and (**d**) particle size distribution curve of sample **2a**.

Heating of **2**, as in the case of **1**, leads to the destruction of its crystal structure and the formation of a two-component system consisting of large blocks and nanosized particles crystallized on their surface (Figure 9).

However, sample **2a** is characterized by a higher degree of destruction of the matrix and the formation of nanosized particles of cobalt monoboride in both agglomerated and non-agglomerated forms (Figure 9b,c). Cobalt monoboride particles in sample **2a** have a spherical shape. The particle sizes are in the range of 2–26 nm, with an average value of 10 nm. According to the size distribution curve, the nanosized particles of sample **2a** are characterized by a smaller size spread.

**Figure 10:**
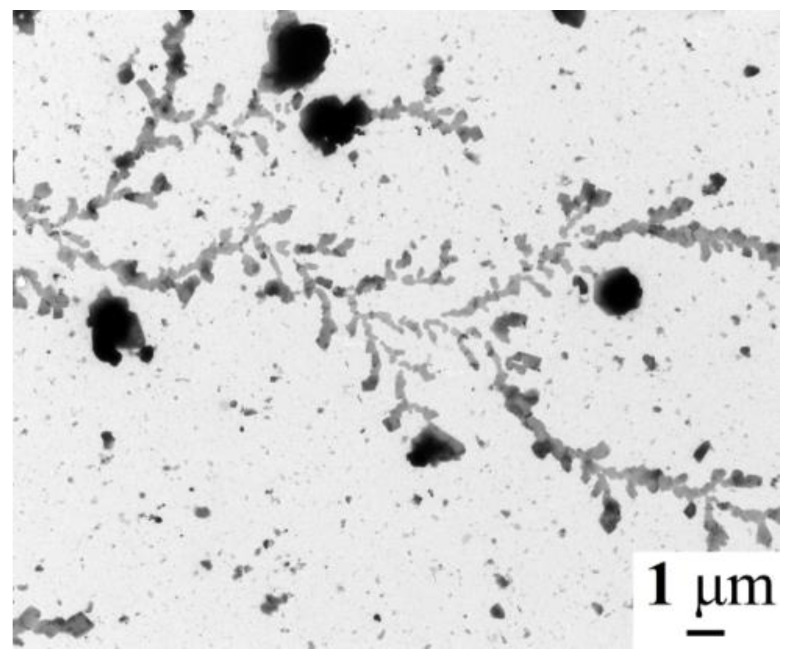
Recrystallization found for sample **3a**.

Heating of **3**, as in the case of **1** and **2**, leads to the destruction of the crystal structure. However, it should be noted that, in all cases, an aqueous suspension was prepared for the study of samples by the TEM method, which was then sprayed onto carbon-coated copper grids. When preparing an aqueous suspension of sample **3a**, a sharp odor was present. The process of dissolution is indirectly confirmed by the observed process of sample recrystallization on a carbon film in the form of separate branches (Figure 10).

**Figure 11:**
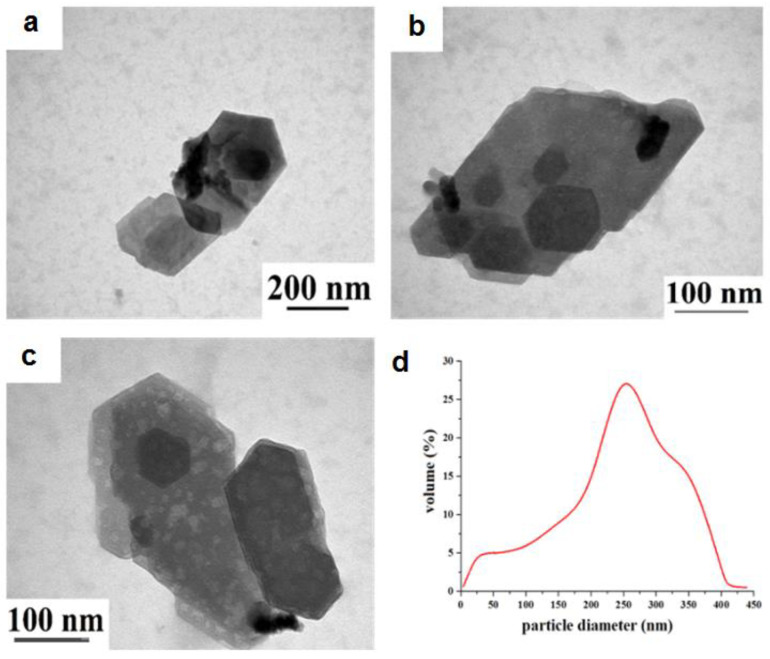
(**a**–**c**) TEM images and (**d**) particle size distribution curve of sample **3a**.

The crystals of sample **3a** have the shape of regular hexagons (Figure 11a–c). It should be noted that sample **3a** is characterized by the formation of crystals with well-defined faces, which indicates the perfection of the structure of the synthesized particles.

At the same time, an intermediate stage of the formation of faces of future crystals with a disordered defect structure is fixed (Figure 11a–c), as evidenced by the inhomogeneity of the structure of the synthesized particles. Figure 11d shows the particle size distribution curve: the particle size is in the range of 25–400 nm with an average size of about 250 nm.

**Scheme 2:**
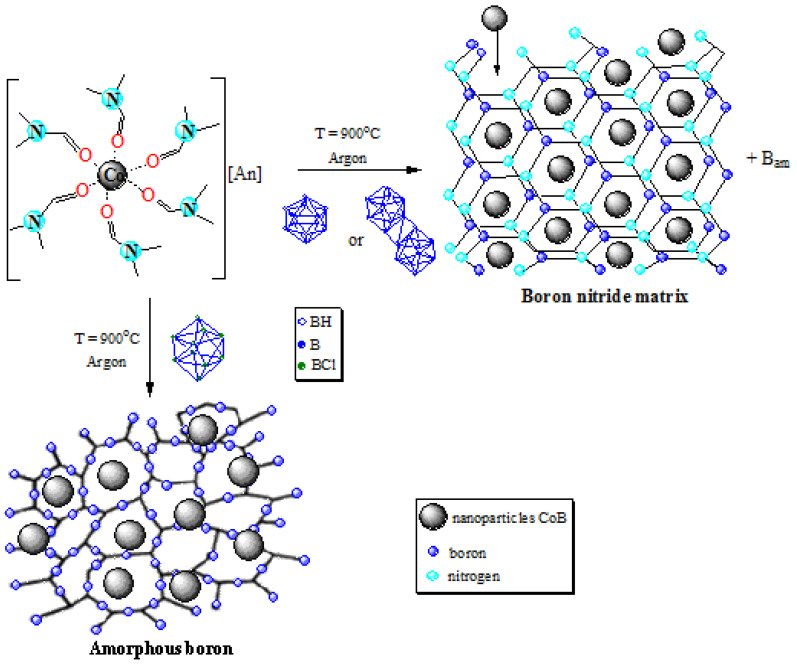
Schematic formation of nanocrystalline cobalt boride during thermolysis of **1**–**3**.

Analyzing the results obtained, the process of formation of nanocrystalline cobalt monoboride in the course of thermal decomposition of cobalt(II) coordination compounds with boron clusters can be represented as follows (Scheme 2).

The absence of BN in **3a** is explained by the fact that the thermolysis of **3** containing [B_10_Cl_10_]^2−^ is accompanied by the destruction of the boron cluster and release of chlorine atoms. The latter, in turn, are a strong oxidizing agent, which leads to the oxidation of the amide nitrogen atom N^3–^, which is formed as a result of the destruction of dimethylformamide molecules, to a neutral N_2_ molecule followed by its removal from the system. In addition, the released chlorine interacts with the boron atoms formed as a result of the destruction of the boron cluster, which leads to the formation of a chlorine-boron compound. It should be noted that chlorine in the composition of annealing product **3a** acts as a mineralizer, which improves the structure of cobalt monoboride particles (Figure 11).

The presence of chlorine in the annealing product **3a** was qualitatively confirmed by X-ray fluorescence analysis. Note that this method qualitatively demonstrated the presence of Co in the thermolysis product **3a**. The presence of analytical lines Cl*K_α_*, Cl*K*_β_, Co*K_α_*, and Co*K*_β_ was revealed with a value many times higher than the background component (Figure S7).

## 3. Experimental

### 3.1. Synthesis of Compounds

*N,N*-dimethylformamide (DMF) and Co(NO_3_)_2_·6H_2_O (≥98.0%) were purchased from Sigma-Aldrich and used without additional purification. Powder amorphous boron (≥95%) was purchased from Merck. (Et_3_NH)_2_[B_12_H_12_] was synthesized from decaborane-14 through the formation of 1,6-bis(triethylamine)decaborane according to the procedure [40,41]. (Et_3_NH)_2_[*trans*-B_20_H_18_] was obtained by oxidation of (Et_3_NH)_2_[B_10_H_10_] with iron(III) chloride in an aqueous solution according to the procedure [42]. (Et_3_NH)_2_[B_10_Cl_10_] was prepared from (Et_3_NH)_2_[B_10_H_10_] by chlorination of aqueous solution of potassium salt of the *closo*-decaborate anion according to the procedure reported [43].

#### 3.1.1. Preparation of [Co(DMF)_6_][An], Where [An] = [B_12_H_12_]^2−^ (Compound **1**) and [trans-B_20_H_18_]^2−^ (Compound **2**∙3H_2_O)

A solution of salt (Et_3_NH)_2_[B_12_H_12_] or (Et_3_NH)_2_[*trans*-B_20_H_18_] (5 mmol) in DMF (10 mL) was added to a solution of Co(NO_3_)_2_·6H_2_O (5 mmol) dissolved in DMF (10 mL). As a result of evaporation in air for 2 weeks, purple crystalline precipitate **1** or **2**∙3H_2_O was obtained, which was filtered off and dried in air. Yield was 59% (for **1**) and 54% (for **2**∙3H_2_O). Single crystals of **1** suitable for X-ray diffraction were obtained directly from the reaction solution.

**1**: Calcd. for CoC_18_H_54_N_6_O_6_B_12_, %: C, 33.82; H, 8.51; N, 13.15; Co, 9.22; B, 20.3. Found, %: C, 33.77; H, 8.57; N, 13.19; Co, 9.16; B, 19.5. IR (NaCl, Nujol, cm^−1^): ν(BH) 2466; ν(C=O)_DMF_ 1651.

**2** (dry sample): Calcd. for CoC_18_H_60_N_6_O_6_B_20_, %: C, 29.54; H, 8.26; N, 11.48; Co, 8.05; B, 29.5. Found, %: C, 29.58; H, 8.20; N, 11.43; Co, 8.12; B, 29.1. IR for **2**·3H_2_O (NaCl, Nujol, cm^−1^): ν(OH) 3450; ν(BH) 2532, 2497; ν(C=O)_DMF_ 1664.

#### 3.1.2. Preparation of [Co(DMF)_6_][B_10_Cl_10_] (**3**)

Compound **3** was synthesized from Co(NO_3_)_2_·6H_2_O and (Et_3_NH)_2_[B_10_Cl_10_] in DMF according to the procedure reported [40]. Yield, 82%.

Calcd. for CoC_18_H_42_N_6_O_6_Cl_10_B_10_, %: C, 22.52; H, 4.41; N, 8.75; Co, 6.14; B, 11.3. Found, %: C, 22.41; H, 4.54; N, 8.65; Co, 6.19; B, 10.9. IR (NaCl, Nujol, cm^−1^): ν(BCl) 1157, 1002, 843; ν(C=O)_DMF_ 1652.

#### 3.1.3. Preparation of Compounds **1a**–**3a**

Compounds **1–3** were annealed in a quartz tube-reactor heated by a resistance furnace and sealed hermetically with tapered silica glass ground joints. The studied samples were placed in corundum crucibles. Annealing was carried out in a high purity argon (5–7 ppm O_2_, 10 ppm H_2_O) flow at a gas flow rate of 300 mL/min and heating at 10 °C/min. The annealing time for each sample was 120 min (in a flow of high purity argon) at 900 °C; the sample was cooled down to room temperature in an Ar flow. Note that with a longer exposure, the phase composition (qualitative and quantitative) did not change. Visually, the thermolysis products **1a**–**3a** are black powders remained stable when treated in air.

### 3.2. Methods of Investigation

Elemental analysis of compounds **1**–**3** for carbon, hydrogen, and nitrogen was performed using a Carlo ErbaCHNS-3 FA 1108 automated elemental analyzer. Boron and metal content was determined on an iCAP 6300 Duo ICP emission spectrometer with inductively coupled plasma. Before the measurements, samples were dried in vacuum to constant weight; for **2**·3H_2_O, solvent-free sample **2** was obtained.

IR spectra of complexes **1**, **2**·3H_2_O, **3** and annealed products **1a**–**3a** and amorphous boron were recorded on a Lumex Infralum FT-02 Fourier-transform spectrophotometer in the range of 4000–600 cm^−1^ at a resolution of 1 cm^−1^. Samples were prepared as Nujol mulls; NaCl pellets were used.

X-ray diffraction study. The single-crystal X-ray diffraction data for **1** performed with a Bruker APEX-II CCD and a Bruker D8 Venture (Centre of Joint Equipment of Kurnakov Institute of General and Inorganic Chemistry, Russian Academy of Sciences) using *φ* and *ω-*scan mode. The data were indexed and integrated using the SAINT program [44]. Absorption correction based on measurements of equivalent reflections (SADABS) were applied [45]. The structures were determined by direct methods and refined by full-matrix least squares technique on *F*^2^ with anisotropic displacement parameters for non-hydrogen atoms. The hydrogen atoms were placed in calculated positions and refined within riding model with fixed isotropic displacement parameters [*U*_iso_(H) = 1.5*U*_eq_(C) for the CH_3_-groups and 1.2*U*_eq_(C) for the other groups]. All calculations were carried out using the SHELXTL program [46] and OLEX2 program package [47]. For details, see Table S1 (Electronic Supporting Information).

The crystallographic data were deposited with the Cambridge Crystallographic Data Center, CCDC no. 2159665. Copies of this information may be obtained free of charge from the Director, CCDC, 12 Union Road, Cambridge CHB2 1EZ, UK (Fax: +44 1223 336033; e-mail: deposit@ccdc.cam.ac.uk or www.ccdc.cam.ac.uk (accessed on 24 November 2022)).

X-ray powder diffraction studies of started samples **1**–**3** and annealed products **1a**–**3a** were performed on a Bruker D8 ADVANCE diffractometer (Cu*K*_α_ radiation, Ni filter, LYNXEYE detector, reflection geometry) in low-background cuvettes with an oriented silicon single crystal substrate in the angle range 2θ = 5°–80° with a step 0.01125°.

X-ray spectral fluorescence analysis of sample **3a** was performed on a wave-dispersive X-ray fluorescence spectrometer SPECTROSCAN MAX (Russia) (voltage 40 kV, current 0.5 mA, X-ray tube anode material Pd, analyzer crystals: LiF(200) and COO_2_. Individual particles were placed between two layers of a polyethylene terephthalate film of thickness 5 µm.

Thermogravimetric analysis (TG-DSC) of samples **1**–**3** was carried out using an SDT Q600 synchronous thermal analyzer in alundum crucibles (40–100 μL) in a flow of high purity argon (100 mL/min) at a sample heating rate of 10 °C/min and within the range temperatures from room temperature to 900 °C.

Samples **1–3** were annealed in a Nabertherm R 30/200/11 tube furnace in an inert gas flow to form samples **1a–3a**. Before annealing, samples were ground in an agate mortar and transferred into thin-walled quartz test tubes, which were then placed in a quartz flow tube at the level of the furnace hot zone.

Transmission electron microscopy (TEM) studies of samples **1a–3a** were carried out on a Jem-1011 instrument at an accelerating voltage of 80 kV. Samples were applied to copper grids by ultrasonic sputtering.

Hirshfeld surface analysis. The Crystal Explorer 17.5 [48] program was used to analyze the interactions within crystal **1**. The donor–acceptor groups are visualized using a standard (high) surface resolution and *d*_norm_ surfaces are mapped over a fixed color scale of −0.640 (red) to 0.986 (blue) a.u.

## 4. Conclusions

Nanocrystalline cobalt boride in the tetragonal modification was prepared by thermal decomposition (900 °C) of preliminarily synthesized cobalt precursors [Co(DMF)_6_][An], where An = [B_12_H_12_]^2−^ (**1**), [B_20_H_18_]^2−^ (**2**) or [B_10_Cl_10_]^2−^ (**3**) in an argon atmosphere. Thermolysis products **1a**–**3a** were identified and characterized by X-ray powder diffraction and IR spectroscopy; X-ray fluorescence data were obtained for **3a**. The average particle size of cobalt monoboride was determined using transmission electron microscopy (TEM). It has been found that the nature of the boron cluster anion affects the phase composition of the thermolysis products (presence of a boron nitride phase during the thermolysis of **1** and **2**), as well as the structural features of the resulting cobalt monoboride (space groups *Pbnm* or *Pnma*). Thermolysis of **1** and **2** afforded CoB particles up to 35 nm in size with a narrow size distribution curve, which is an important task in catalysis; 3 gave CoB with 200–350 nm in size.

## Data Availability

Not applicable.

## Supplementary Materials

The following supporting information can be downloaded at: https://www.mdpi.com/article/10.3390/molecules28010453/s1, Table S1, Crystal data and structure refinement for complex **1**; Figure S1, IR spectra of compounds (a) **1**, (b) **2**·3H_2_O, and (c) **3**; Figure S2, Projection of structure of complex **1** along the *a* axi; Figure S3, Calculated (black) and experimental (red) X-ray powder diffraction patterns for (a) compound **1**, (b) compound **2**, and (c) compound **3**; Figure S4, IR spectra of (a) complex **2**∙*n*H_2_O (blue), sample **2a** (green) and amorphous boron (red)); Figure S5, Fragments of IR spectra of complex **1** (blue), sample **1a** (purple) and amorphous boron (red); Figure S6, Fragments of IR spectra of complex **3** (purple), sample **3a** (red) and amorphous boron (green); Figure S7, X-ray spectral fluorescence spectrum of sample **3a**: (A) determination of Cl, (B) determination of Co.
