# Supplementary material for: A New Approach to the Synthesis of Nanocrystalline Cobalt Boride in the Course of the Thermal Decomposition of Cobalt Complexes [Co(DMF)6]2+ with Boron Cluster Anions
Source: Molecules. 2023 Jan 3;28(1):453. doi: 10.3390/molecules28010453 (PMC9823307; doi:10.3390/molecules28010453)
Supplement: Supplementary file 1 [file molecules-28-00453-s001.zip › molecules-2103628-supplementary.pdf]

SUPPLEMENTARY INFORMATION

for

A new approach to the synthesis of nanocrystalline cobalt boride in the course of the thermal reduction of cobalt complexes with boron cluster anions

Elena A. Malinina, Ivan I. Myshletsov, Grigorii A. Buzanov, Alexey S. Kubasov, Irina I. Kozerozhets, Lyudmila V. Goeva, Svetlana E. Nikiforova, Varvara V. Avdeeva, Konstantin Yu. Zhizhin, Nikolay T. Kuznetsov

Table S1. Crystal data and structure refinement for complex **1**.

Compound	**1**
Empirical formula	$C_{18}H_{54}B_{12}CoN_6O_6$
Formula weight	639.32
Temperature/K	150.15
Crystal system	triclinic
Space group	P-1
a/Å	9.5857(5)
b/Å	18.9576(11)
c/Å	19.1748(11)
α/°	89.9010(10)
β/°	85.0580(10)
γ/°	89.9280(10)
Volume/Å^3	3471.5(3)
Z	4
ρ_{calc}g/cm^3	1.223
μ/mm^{-1}	0.535
F(000)	1356.0
Radiation	MoKα (λ = 0.71073)
Index ranges	$-13 \leq h \leq 13$<br>$-25 \leq k \leq 25$<br>$-25 \leq l \leq 26$
Reflections collected	38452
Independent reflections	18096<br>[R_{int} = 0.0531, R_{sigma} = 0.0906]
Data/restraints/parameters	18096/0/770
Goodness-of-fit on F^2	0.984
Final R indexes [I>=2σ (I)]	R_1 = 0.0510, wR_2 = 0.1001
Final R indexes [all data]	R_1 = 0.1092, wR_2 = 0.1201

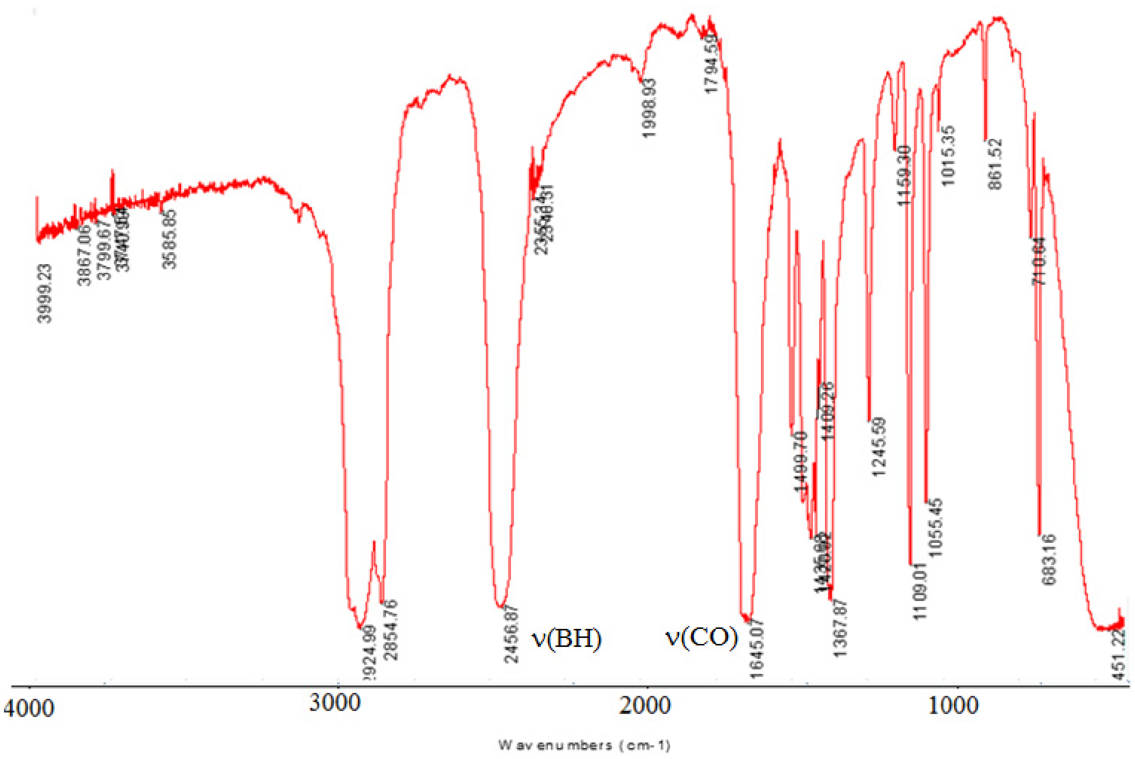

3999.23
3867.06
3799.67
3585.85
2854.76
2456.87
ν(BH)
ν(CO)
1998.93
1794.59
1645.07
1499.70
1409.26
1367.87
1245.59
1159.30
1109.01
1055.45
1015.35
861.52
710.64
683.16
451.22
4000
3000
2000
1000
Wavenumbers (cm-1)

(a)

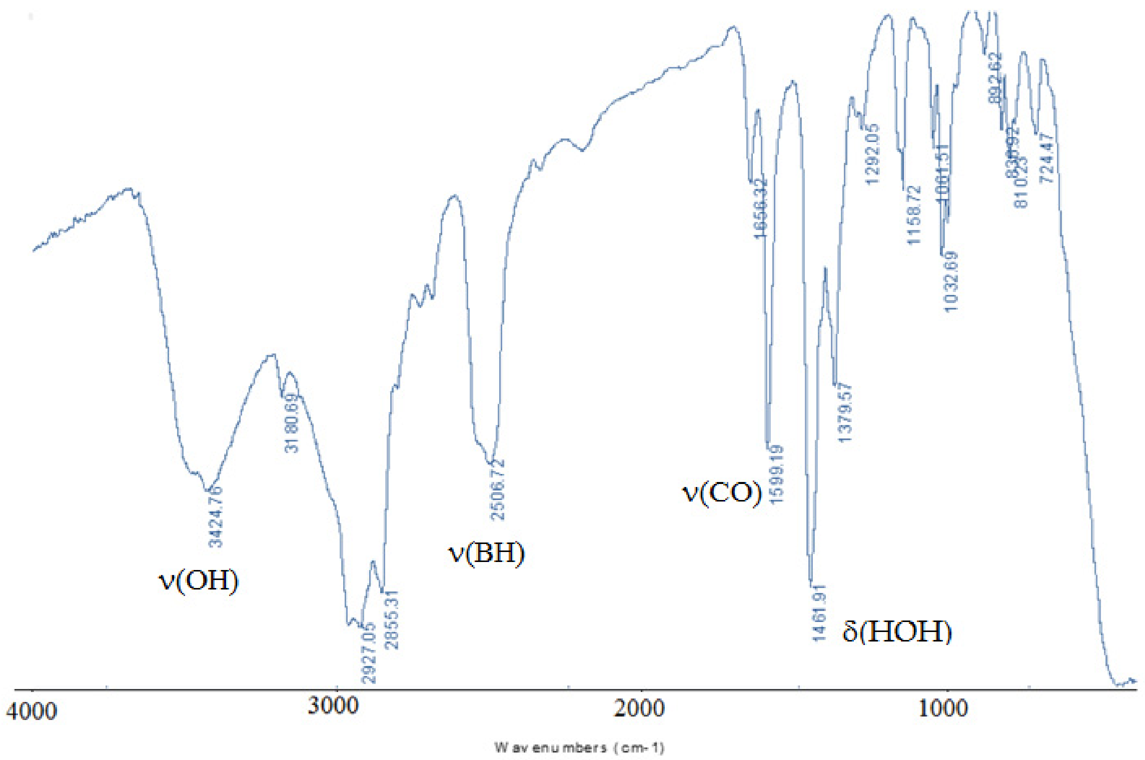

3424.76
3180.69
ν(OH)
2927.05
2855.31
2506.72
ν(BH)
ν(CO)
1656.32
1599.19
1461.91
δ(HOH)
1379.57
1292.05
1158.72
1061.51
1032.69
892.62
810.23
724.47
4000
3000
2000
1000
Wavenumbers (cm-1)

(b)

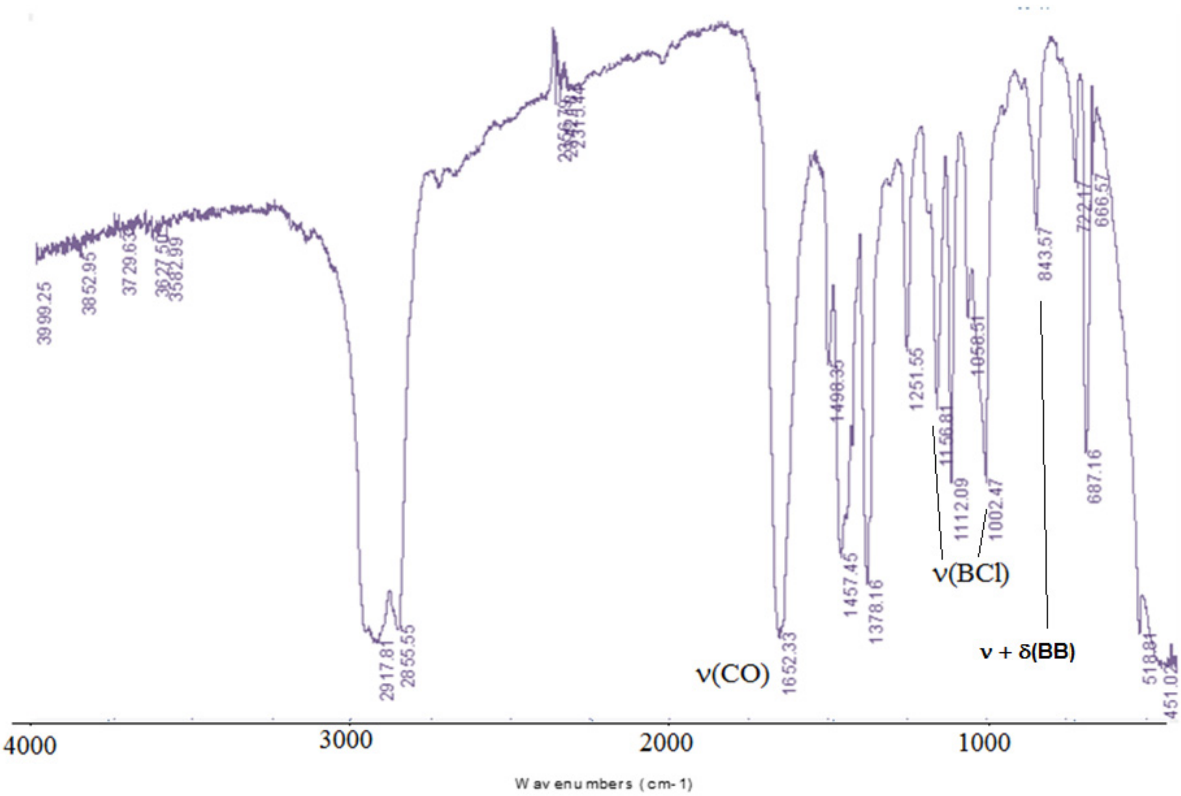

(c)

Figure S1. IR spectra of compounds (**a**) **1**, (**b**) **2**·3H_2O, and (**c**) **3**.

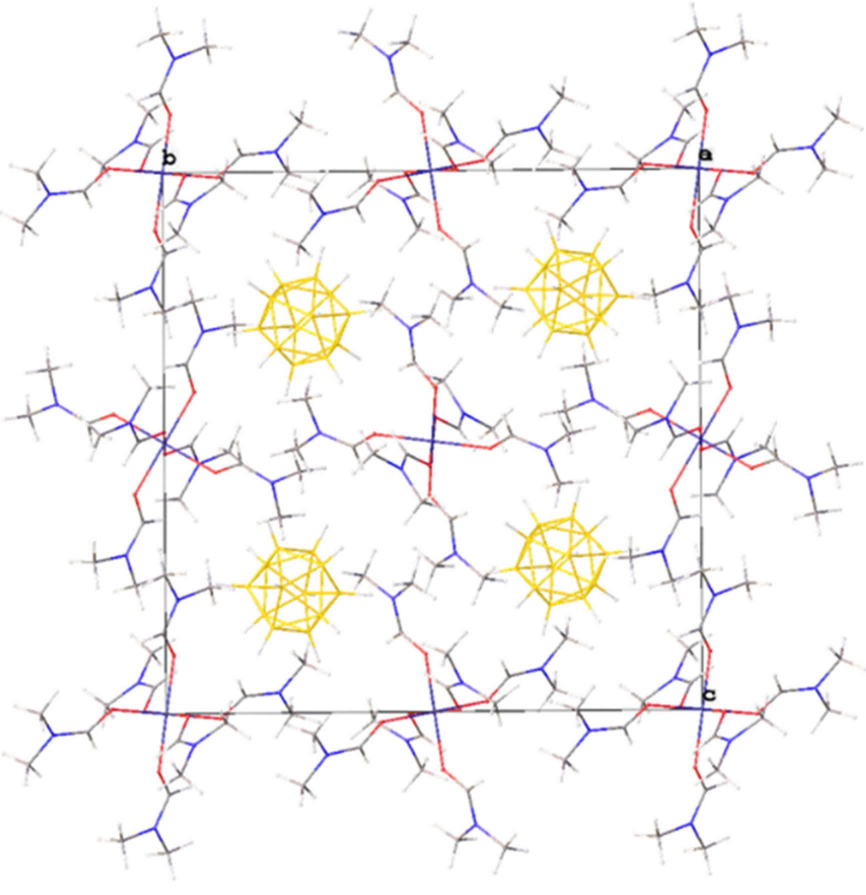

Figure S2. Projection of structure of complex **1** along the *a* axis.

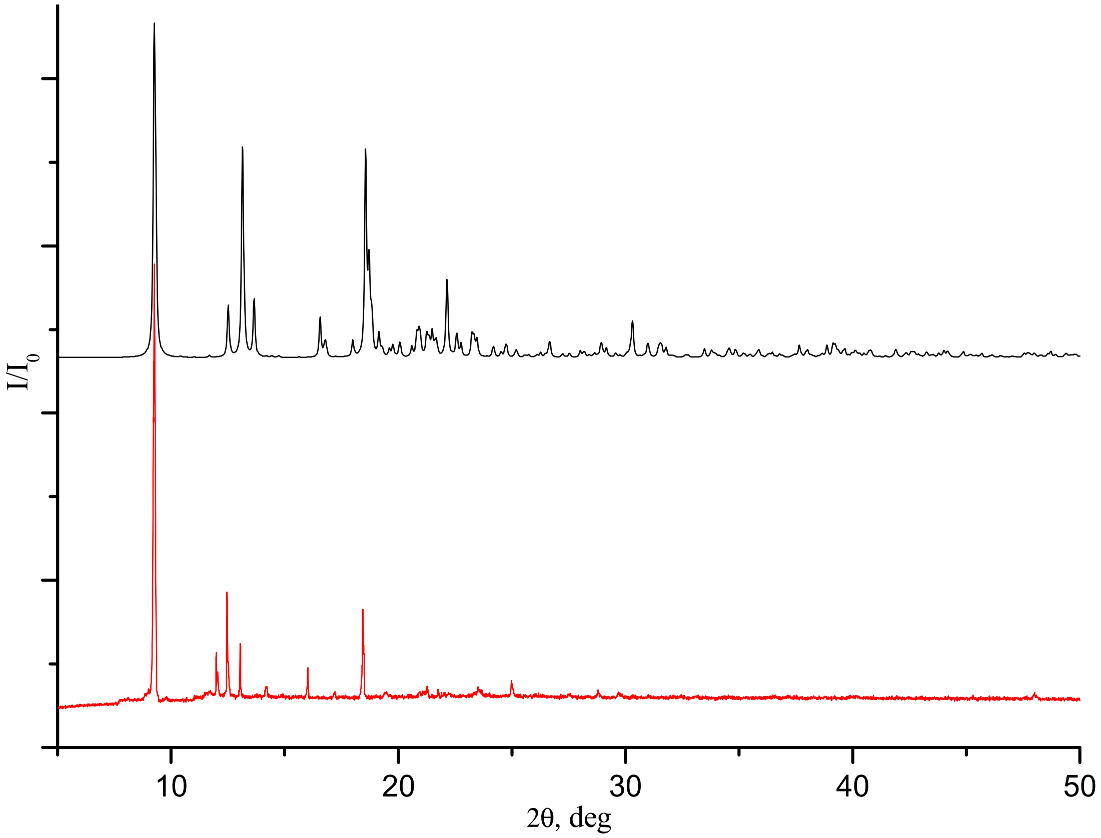

(a)

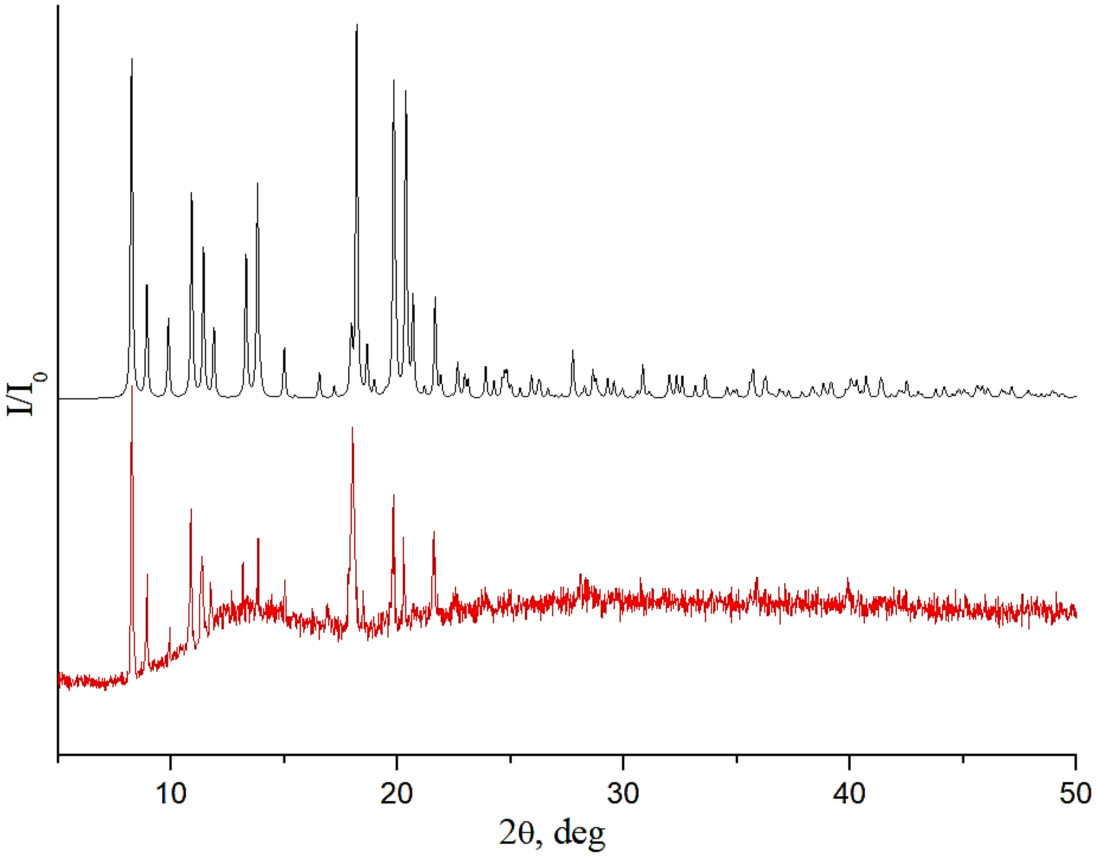

(b)

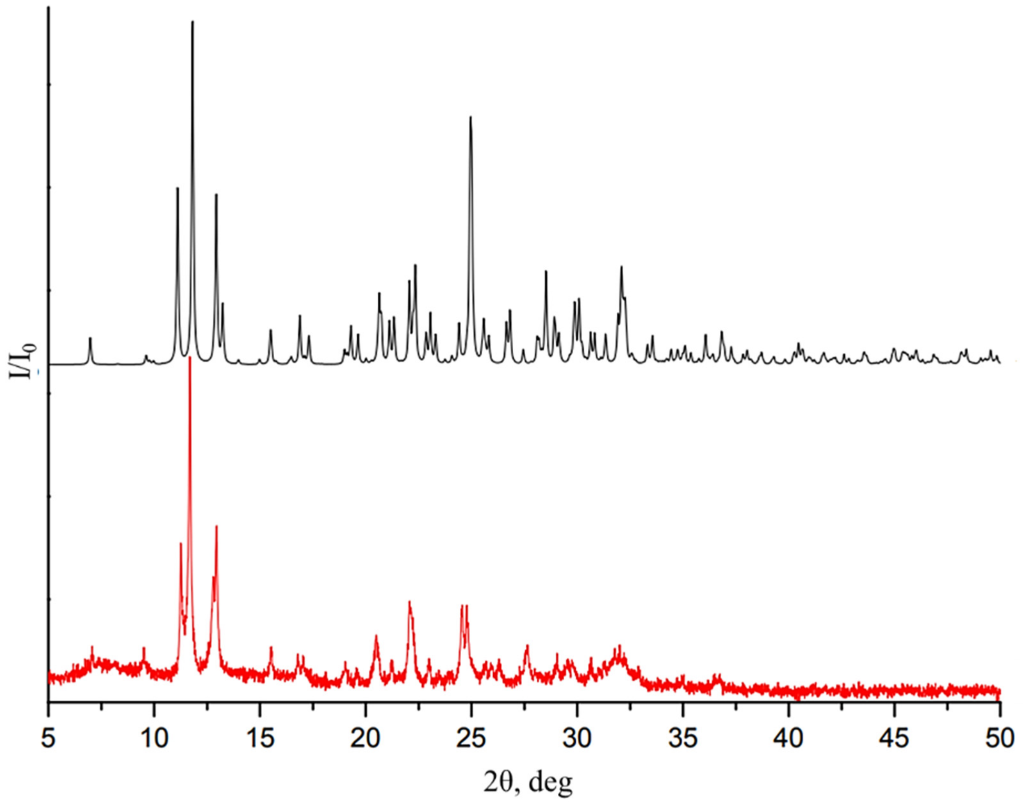

(c)

Figure S3. Calculated (black) and experimental (red) X-ray powder diffraction patterns for (**a**) compound **1**, (**b**) compound **2**, and (**c**) compound **3**.

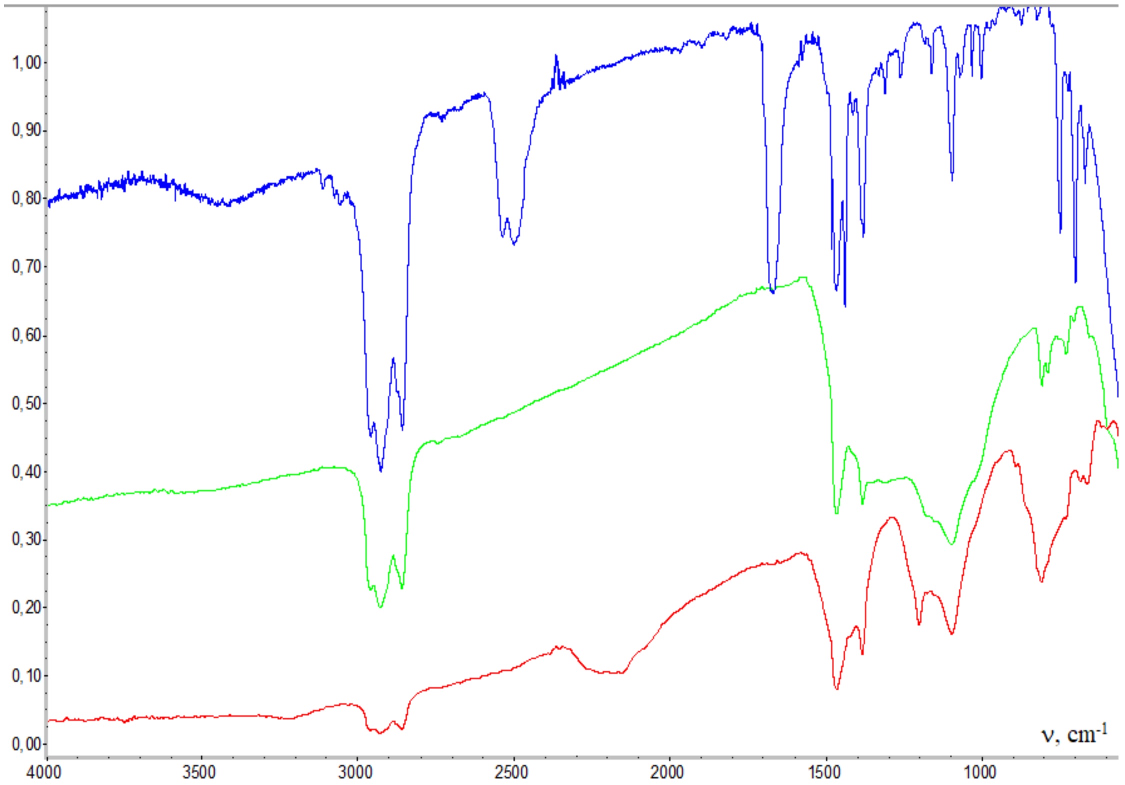

Figure S4. IR spectra of complex **2**·*n*H_2O (blue), sample **2a** (green) and amorphous boron (red).

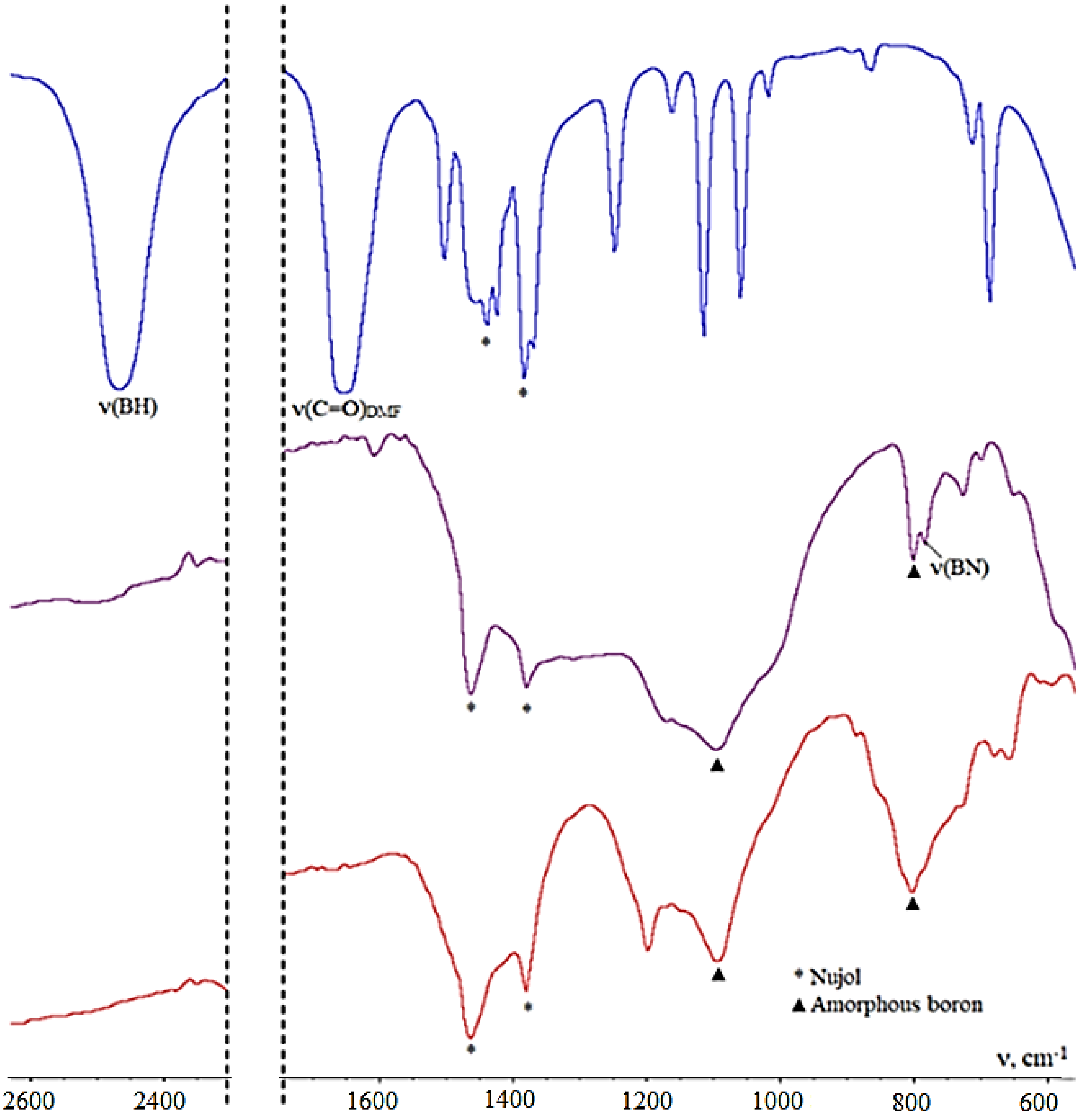

Figure S5. Fragments of IR spectra of complex **1** (blue), sample **1a** (purple) and amorphous boron (red).

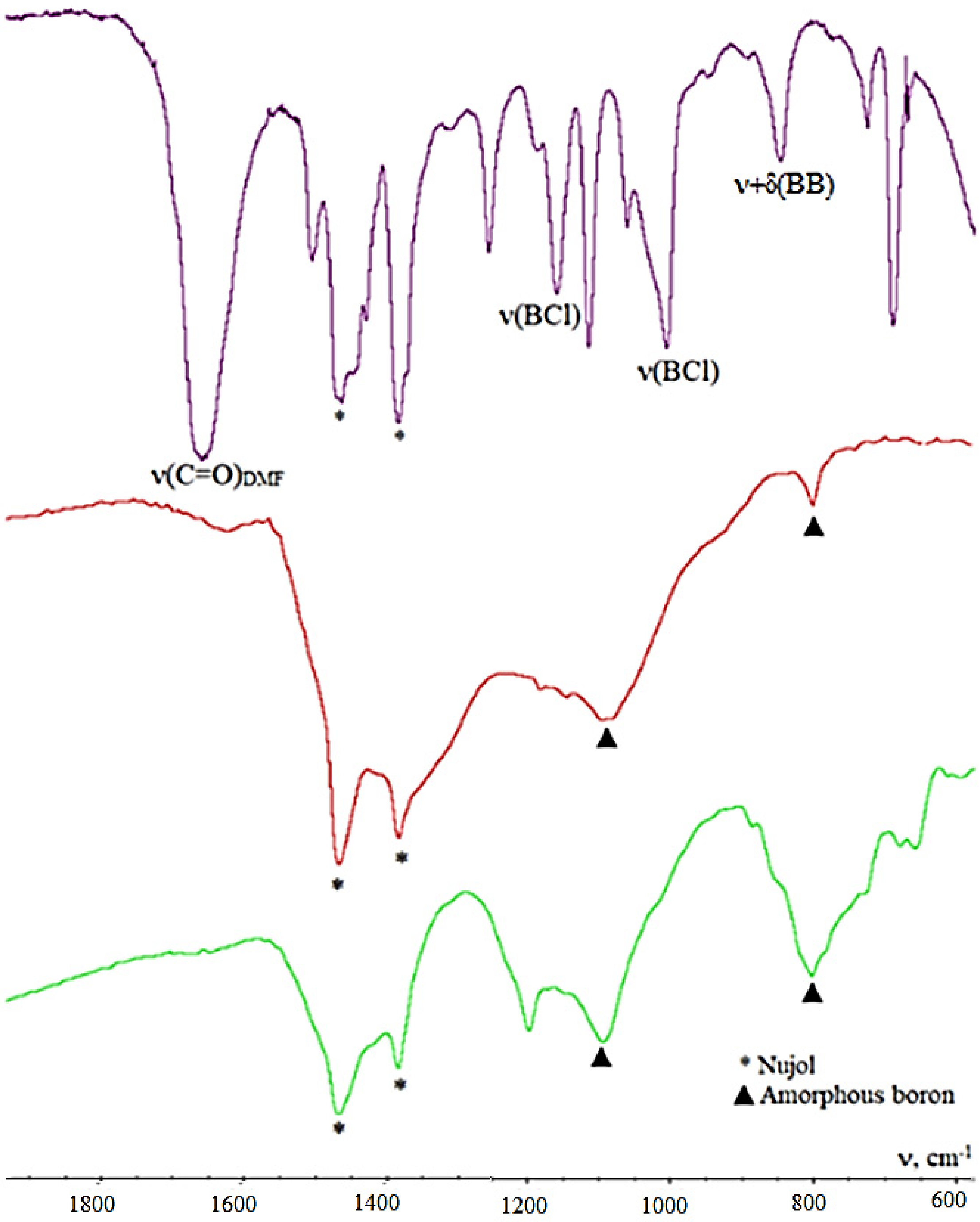

Figure S6. Fragments of IR spectra of complex **3** (purple), sample **3a** (red) and amorphous boron (green).

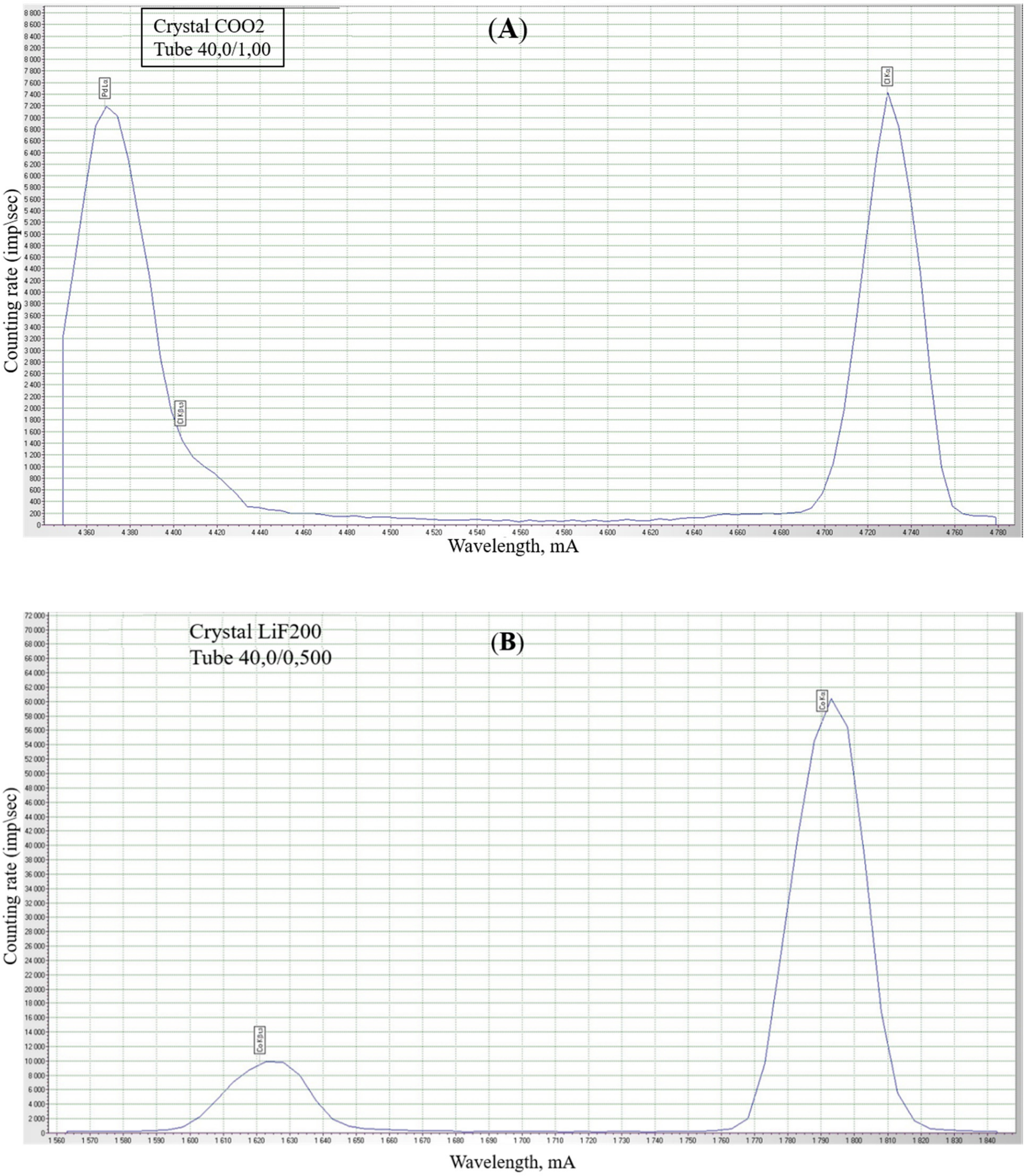

Figure S7. X-ray spectral fluorescence spectrum of sample **3a**: (**A**) determination of Cl, (**B**) determination of Co.
